# Early Postreperfusion Proteomics Reveal Divergent Inflammatory Responses in Kidney Transplantation With Implications on Outcomes

**DOI:** 10.1097/TP.0000000000005561

**Published:** 2025-10-31

**Authors:** Gabriel Strandberg, Carl Raihle, Bo Nilsson, Carl M. Öberg, Anna M. Blom, Sara Axman, Oleg Slivca, Clara Paul, David Berglund, Ali-Reza Biglarnia

**Affiliations:** 1 Department of Surgery, Skåne University Hospital, Malmö, Sweden.; 2 Department of Clinical Sciences Malmö, Lund University, Malmö, Sweden.; 3 Department of Immunology, Genetics and Pathology (IGP), Rudbeck Laboratory C5:3, Uppsala University, Uppsala, Sweden.; 4 Department of Nephrology, Skåne University Hospital, Lund, Sweden.; 5 Department of Clinical Sciences Lund, Lund University, Lund, Sweden.; 6 Department of Translational Medicine, Lund University, Malmö, Sweden.

## Abstract

**Background.:**

Ischemia/reperfusion injury is an unavoidable consequence of kidney transplantation, yet the characteristics of the immediate immune response after reperfusion and its impact on transplant outcomes remain poorly characterized in the clinical setting.

**Methods.:**

We conducted a cohort study including 63 kidney transplant recipients (26 living-donor, 37 deceased-donor) with an extended 4-y follow-up to characterize early postreperfusion inflammatory dynamics and their association with transplant outcomes. Using high-throughput proteomics, we profiled 92 inflammatory markers in the early reperfusion stage. Intraoperative blood samples were collected systemically at baseline and from the transplant vein at 1, 10, and 30 min postreperfusion.

**Results.:**

Our analysis revealed a pronounced early immune response on reperfusion, with distinct inflammatory trajectories between living- and deceased-donor kidney allografts. Living-donor allografts showed proteomic patterns suggestive of a regulatory response, whereas deceased-donor allografts exhibited patterns associated with a cell injury-related response. Notably, interleukin-33 and hepatocyte growth factor were associated with delayed graft function, whereas hepatocyte growth factor also correlated with long-term allograft dysfunction.

**Conclusions.:**

These findings underscore the potential of assessing early postreperfusion inflammation to improve clinical risk stratification and guide future biomarker validation efforts.

## Graphical abstract

**Figure:**
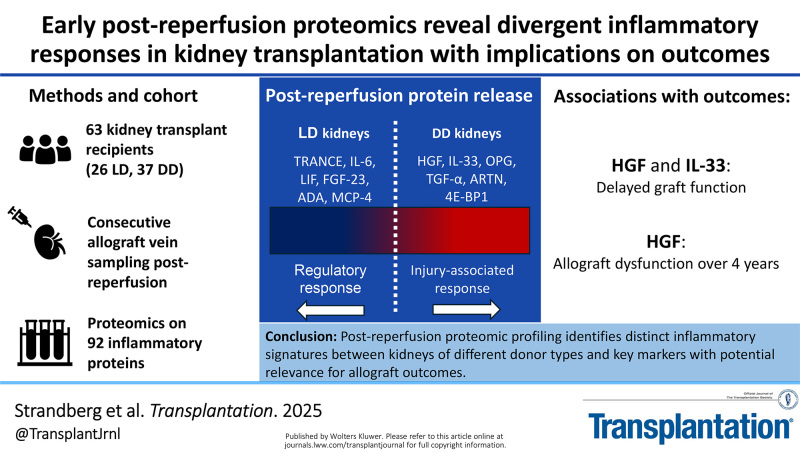

## INTRODUCTION

Kidney transplantation (KT) remains the treatment of choice for patients with end-stage renal disease, offering substantial gains in both survival and quality of life.^1,2^ However, the success of KT is challenged by injuries that impair allograft function, with ischemia/reperfusion injury (IRI) being a primary and inevitable contributing factor.^3,4^ This injurious response arises when oxygen supply and blood flow are restored to allografts after a period of ischemia, orchestrating a complex inflammatory response that can lead to both early and long-term graft dysfunction.^4-7^

Recently, we demonstrated that IRI initiates a coordinated activation of cascade systems within the intravascular innate immune response, with downstream effects on allograft performance at both short- and long-term functions. Specifically, we identified soluble C5b-9 (sC5b-9), a traditional marker of complement activation, as a response marker for thromboinflammation due to its strong associations with activation markers of the coagulation and kallikrein-kinin cascades within the intravascular innate immune system. Importantly, this thrombo-inflammatory response to IRI differed by donor type, being prominent in the deceased-donor (DD)-KT population while largely absent in the living-donor (LD)-KT population.^8^

In this exploratory study, we applied high-throughput unbiased proteomic profiling using the Olink Inflammation Panel, which targets 92 proteins involved in various inflammatory pathways, to further characterize the early inflammatory response to IRI in a well-characterized cohort of KT recipients with 4-y follow-up. To enhance sampling precision, we collected samples directly from the transplant vein immediately postreperfusion, allowing for precise characterization of localized inflammatory dynamics that are often diluted or delayed in systemic circulation. Our aim was to identify specific inflammatory signatures in kidneys derived from different donor types and evaluate their associations with allograft function, with the broader goal of uncovering novel protein markers relevant to IRI and transplant outcomes.

## MATERIALS AND METHODS

### Study Population

The study included 63 consecutive KT recipients (26 from LDs and 37 from DDs) transplanted between August 2018 and June 2019 at the Department of Transplantation, Skåne University Hospital, Sweden. All LD kidneys and 22 DD kidneys were preserved with static cold storage, whereas the remaining 15 DD kidneys were preserved with nonoxygenated pulsatile hypothermic machine perfusion (LifePort Kidney Transporter, Itasca, IL). All DD kidneys were procured from donation-after-brain-death donors.

Patients received a maintenance immunosuppressive regimen combining tacrolimus, mycophenolate mofetil, and prednisolone according to the SYMPHONY protocol.^9^ Per standard, 61 patients (96.8%) received induction therapy with basiliximab and methylprednisolone. One DD-KT recipient received rituximab due to ABO-incompatibility and 2 others received thymoglobulin due to preexisting donor-specific antibodies. Pretransplant immunosuppression was administered according to a standardized regimen, with oral agents (excluding prednisolone) given in the immediate preoperative phase and induction therapy initiated after insertion of a central line upon the patient’s arrival in the operating room.

Data on donor and recipient characteristics, along with key outcomes such as plasma creatinine, estimated glomerular filtration rate (eGFR), delayed graft function (DGF), de novo donor-specific antibodies, graft failure, patient death, and biopsy-proven acute rejections of any type after indication biopsies, were collected from patient records, the local transplant registry, and the ScandiaTransplant database for a total follow-up of 48 mo posttransplantation. DGF was defined as the need for dialysis within the first week posttransplantation. The Lund-Malmö revised formula^10^ was used to calculate eGFR levels (mL/min/1.73 m^2^). The study was approved by the Regional and National Ethical Committee (DNR 2017-79), and written informed consent was obtained from all patients. Baseline characteristics are summarized in Table 1.

**TABLE 1.** Baseline characteristics of the study population by donor type subgroups

Baseline characteristics	LD-KT (N = 26)	DD-KT (N = 37)
Recipient		
Sex, male	18 (69.2)	26 (70.3)
Age, y	45.5 (36.0–54.0)	55.0 (46.0–62.0)
BMI	25.9 (22.9–27.6)	25.1 (23.8–27.7)
Donor		
Sex, male	11 (42.3)	19 (51.4)
Age	51.0 (46.0–58.0)	58.0 (50.0–67.0)
BMI	26.4 (23.6–28.6)	24.7 (22.2–29.3)
Kidney donor risk index	NA	1.5 (1.3–1.97)
Cold ischemic time	116.5 (96.0–154.0)	692.0 (547.0–902.0)
Preformed DSA	4 (15.4)	6 (16.2)
Graft preservation method		
Static cold storage	26 (100.0)	22 (59.5)
Hypothermic machine perfusion	0 (0.0)	15 (40.5)
Pretransplant dialysis status		
Preemptive	11 (42.3)	4 (10.8)
Hemodialysis	9 (34.6)	24 (64.9)
Peritoneal dialysis	6 (23.1)	9 (24.3)
Days of pretransplant dialysis	392 (182–730)	1074 (630–1642)
Induction therapy		
Basiliximab	26 (100.0)	34 (91.9)
Basiliximab and rituximab	0 (0.0)	1 (2.7)
Thymoglobulin	0 (0.0)	2 (5.4)
Cause of kidney failure		
Glomerulonephritis	14 (53.8)	11 (29.7)
Diabetic nephropathy	3 (11.5)	6 (16.2)
Hypertensive nephrosclerosis	3 (11.5)	5 (13.5)
Polycystic kidney disease	3 (11.5)	4 (10.8)
Other	2 (7.7)	4 (10.8)
Alport’s syndrome	1 (3.8)	3 (8.1)
Unknown	0 (0.0)	4 (10.8)

Categorical variables are presented as frequencies (percentages) and continuous variables as median (first–third quartile). *P* values are expressed for comparisons of the 2 groups.

BMI, body mass index; DD, deceased-donor; DSA, donor-specific antibody; KT, kidney transplantation; LD, living-donor.

### Sample Collection

Six milliliter whole-blood samples were drawn by venipuncture with a butterfly needle system (21G BD Vacutainer UltraTouch, Becton, Dickinson and Company) into EDTA vacutainer tubes (K2E BD Vacutainer, Becton, Dickinson and Company). A baseline sample was drawn from the recipient’s external iliac vein before allograft implantation, followed by consecutive sampling from the renal transplant vein at 1, 10, and 30 min postreperfusion. Immediately after collection, samples were cooled in ice-sludge, followed by centrifugation at 1900*g* for 10 min at 4 °C to separate the plasma, which was aliquoted and stored at –80 °C.

### Samples Analysis

Protein quantification was performed using a proximity extension assay with the Olink Proteomics–Inflammation Panel, including 92 target proteins listed in **Table S1** (**SDC,** https://links.lww.com/TP/D323). Output on quantified protein levels was received in arbitrary normalized protein expression (NPX) units on a log2 scale and then transformed to a linear scale.

All samples were anonymized with numeric codes and organized in a random order. The laboratory staff from Olink Proteomics were kept unaware of the study design and purpose. The assay platform has been evaluated for specificity, sensitivity, dynamic range, and reproducibility across both healthy and diseased plasma and serum samples, with detailed validation reports available from the manufacturer (Olink Proteomics, Validation methods and results: https://olink.com/knowledge/documents).

### Statistical Analysis

Given the larger number of simultaneously assessed proteins (n = 92) relative to the study population (n = 63), subgrouping was restricted to transplant cases by donor type (LDs and DDs) to retain statistical power. Postreperfusion protein levels were subtracted from their baseline values to normalize for preexisting inflammation. Continuous variables are expressed as medians (first–third quartile) and categorical variables as frequencies (percentages). Protein quantities are presented in arbitrary NPX values. Mann-Whitney *U* tests and chi-square/Fisher exact tests were used to assess differences in characteristics and general outcome parameters, with a significance threshold at a *P* value of <0.05. For initial screening of proteins, repeated Mann-Whitney *U* tests with Holm-Šídák corrections were performed on all 92 markers to compare protein levels between the DD- and LD-KT-groups. An initial significance threshold at a *P* value of <0.05 was used to assess differences between KT cases by donor type, followed by a stricter threshold at a *P* value of <0.01 to select upregulated proteins for outcome analyses. ANOVA on aligned rank-transformed data with Geisser-Greenhouse correction was used to assess variation of protein levels by donor type and time postreperfusion, including interactions.

Proteins upregulated, with a *P* value of <0.01, in the subgroup comparison were integrated into a single continuous parameter through area under the curve (AUC) calculations on baseline-subtracted 1-, 10-, and 30-min levels postreperfusion for each patient, capturing areas above preimplantation baseline levels. Two patients (both LD-KTs) had missing values for all proteins at 1 timepoint: one at 10 min and the other at 30 min. Their omission did not affect downstream results, and they were therefore included in the analysis as available. Associations between protein AUC levels and DGF were assessed using a receiver operating characteristic (ROC) analysis with Bonferroni correction and Mann-Whitney *U* tests. Long-term graft function was represented by the 4-y AUC of eGFR (mL/min/1.73 m² × months) calculated from 1, 3, 6, 12, 24, 36, and 48 mo posttransplantation. Multiple linear regression was used to assess associations between protein AUC levels and long-term graft function, applying log-transformations on individual protein AUC levels to meet the normality assumptions, stabilize variance, and improve model fit.

To investigate the effect of cold ischemic time (CIT) on postreperfusion inflammation, K-means clustering (k = 2) was used to identify clusters in protein levels by elapsed CIT, followed by ROC analysis with Youden’s J Statistic to estimate the CIT threshold associated with increased protein release.

Data are presented as tables, volcano plots, and scatter plots. Statistical analyses were performed using IBM SPSS Statistics 28 (IBM Corp, Armonk, NY), and GraphPad Prism version 10.3.1 (GraphPad Software, San Diego, CA).

### Guidelines

This work complies with the Strengthening the Reporting of Observational Studies in Epidemiology Guideline for cohort studies.^11^

## RESULTS

### Characteristics and General Clinical Outcomes

Baseline donor and recipient characteristics are summarized in Table 1.

There was no significant difference in CIT between nonoxygenated hypothermic machine perfused and static cold-stored DD kidneys (684.0 [interquartile range [IQR], 546.8–858.0] and 781.0 [IQR, 504.0–1029.0], respectively, *P* = 0.53). Among DD-KT recipients, 6 cases of DGF occurred. There were 2 cases of patient death with functioning grafts in the DD-KT group: one on day 64 due to tissue-invasive cytomegalovirus disease and another on day 939 from an undocumented cause. One patient in the DD-KT group died with concurrent graft failure on day 774, due to COVID-19 infection and multiorgan failure. Additionally, there were 3 cases of graft failure in the DD-KT group due to acute antibody-mediated rejection, recurrence of thrombotic microangiopathy, and unknown primary cause on days 151, 396, and 1397, respectively. In LD-KT, 1 case of graft failure occurred because of the recurrence of focal segmental glomerulosclerosis on day 469 posttransplantation. One patient each from the LD- and DD-KT groups was lost to follow-up due to emigration on day 69 and 1100, respectively.

At the 48-mo follow-up, there were no differences between LD- and DD-KT groups in terms of overall transplant function, graft and patient survival, or incidence of biopsy-proven acute rejections (Table 2).

**TABLE 2.** Outcome parameters of the study population separated by LD- and DD kidney transplant recipients

Outcomes by 48 mo posttransplantation	Donor type (N = 63)		*P*
	LD (N = 26)	DD (N = 37)	
P-Creatinine, µmol/L	111.5 (91.5–132.0)	121.0 (92.0–147.0)	0.41
eGFR, mL/min/1.73 m^2^	58.0 (42.0–69.0)	51.0 (36.0–64.0)	0.29
Non-death-censored graft failure	1 (3.8)	6 (16.2)	0.22
Delayed graft function	0 (0.0)	6 (16.2)	0.038
Patient death	0 (0.0)	3 (8.1)	0.26
Biopsy-proven acute rejection	8 (30.8)	10 (27.0)	0.78
De novo DSA	3 (11.5)	4 (10.8)	1.00

Data are presented as frequencies (column percentages) and medians (first–third quartile). *P* values are expressed for comparisons of the 2 groups.

DD, deceased-donor; DSA, donor-specific antibody; eGFR, estimated glomerular filtration rate; LD, living-donor.

### Postreperfusion Protein Release by Donor Type

Across the study cohort, we first screened all 92 markers using a standard statistical threshold of a *P* value of <0.05 to identify significantly increased proteins during the postreperfusion phase between KT cases of different donor types. From this initial screening, only markers that exhibited distinct upregulation with a more stringent *P* value of <0.01 were subsequently evaluated for their association with clinical outcomes. This stepwise approach ensured that only the most robustly upregulated markers were prioritized for outcome analyses, minimizing the risk of overinterpretation and enhancing the reliability and clinical relevance of the findings.

Immediately after reperfusion, the DD-KT group showed an increased release of hepatocyte growth factor (HGF), osteoprotegerin (OPG), artemin (ARTN), and eukaryotic translation initiation factor 4E-binding protein 1 (4E-BP1) at 1 min compared with LD-KT (*P* < 0.001, *P* < 0.001, *P* < 0.001, and *P* = 0.022, respectively). The increased release of HGF in DD-KT persisted throughout the postreperfusion sampling period at 1, 10, and 30 min (*P* < 0.001, *P* < 0.001, and *P* = 0.0013). Additionally, transforming growth factor alpha (TGF-α) and interleukin-33 (IL-33) levels rose in the DD-KT group from 10 to 30 min postreperfusion (*P* < 0.001, *P* = 0.0038, and *P* = 0.0011, *P* < 0.001, respectively).

**FIGURE 1.**
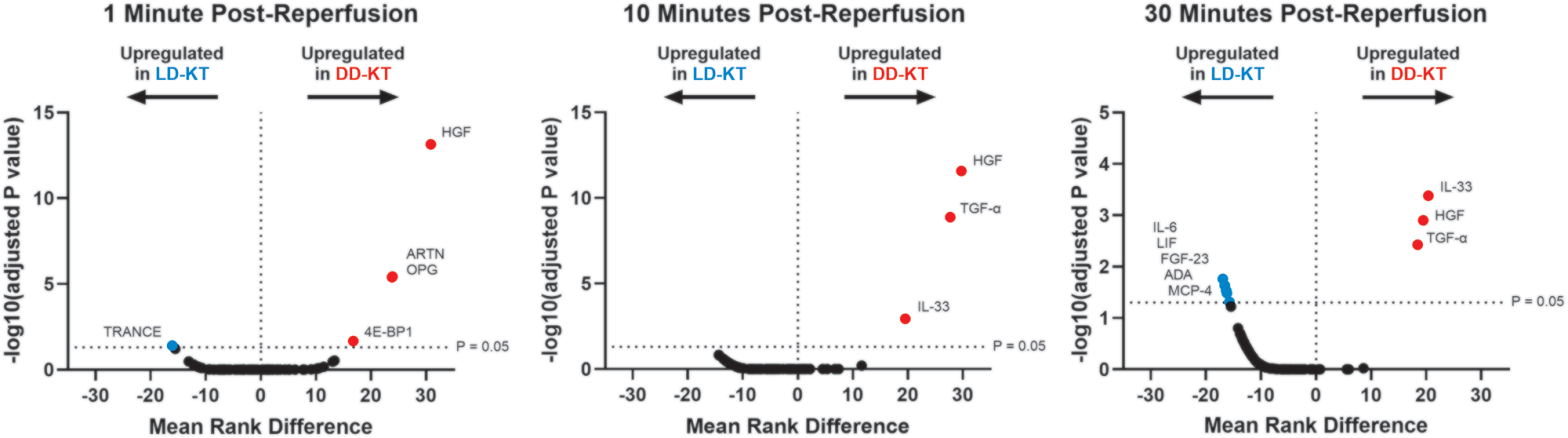
Postreperfusion inflammatory protein expression profiles by donor type. Volcano plots presenting the –log10 adjusted *P* values and mean rank differences from Mann-Whitney *U* tests on all 92 proteins between donor types of transplant cases at 1, 10, and 30 min postreperfusion. Mean rank differences are presented as DD kidneys minus LD kidneys. Red and blue dots indicate upregulated proteins in DD and LD kidneys, respectively. A horizontal dotted line indicates the –log10 adjusted *P* value for *P* = 0.05. Proteins above the significance threshold are labeled. DD-KT, deceased-donor kidney transplantation; LD-KT, living-donor kidney transplantation.

In the LD-KT group, an immediate release of tumor necrosis factor-related activation-induced cytokine (TRANCE) was observed at 1 min postreperfusion (*P* = 0.039). Additionally, IL-6, leukemia inhibitory factor (LIF), fibroblast growth factor 23 (FGF-23), adenosine deaminase (ADA), and monocyte chemoattractant protein 4 (MCP-4) levels were elevated in LD-KT at 30 min (*P* = 0.017, *P* = 0.023, P = 0.029, *P* = 0.033, and *P* = 0.049, respectively; Figure 1).

Results from the multiple comparison analyses for all 92 markers are provided in **Table S1** (**SDC,** https://links.lww.com/TP/D323).

As a complementary analysis, differences in protein release between preservation methods of DD kidneys were also assessed. In kidneys preserved in nonoxygenated hypothermic machine perfusion, increased 30-min release of C-X-C motif ligand 1, ADA, and IL-7 was observed compared with cold-stored kidneys (*P* = 0.018, *P* = 0.041, and *P* = 0.046, respectively). However, no differences were observed at 1 and 10 min postreperfusion (**Table S2, SDC,** https://links.lww.com/TP/D323).

### Temporal Protein Release Dynamics between Kidneys of Different Donor Types

**FIGURE 2.**
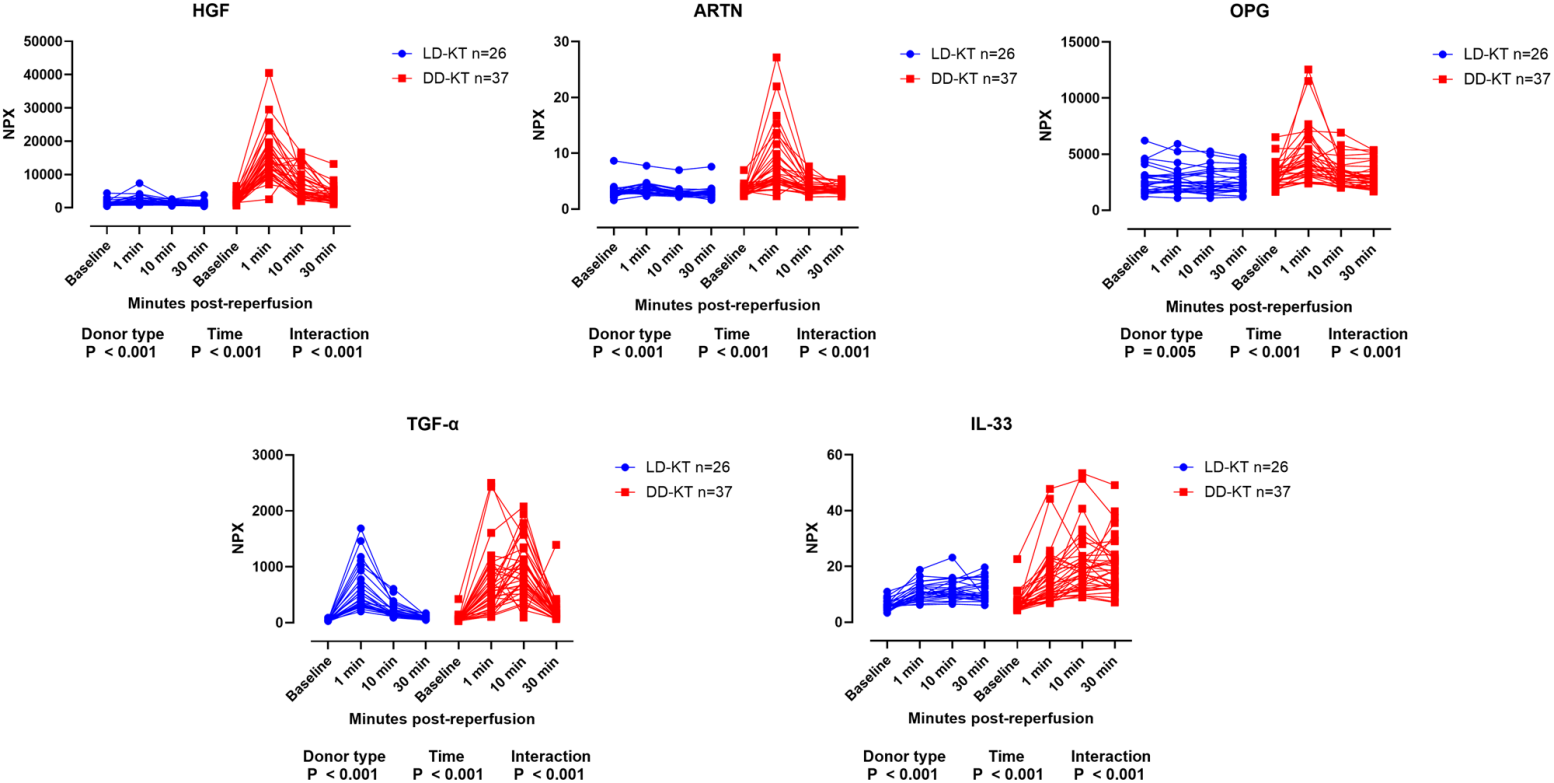
Protein levels by donor type and sampling time. Connected scatter plots of protein levels by sampling time and donor type. Protein levels are presented as nonbaseline adjusted to allow for visualization of preimplantation levels. Blue and red denote LD-KT and DD-KT, respectively. Proteins below the alpha limit of 0.01 from the multiple comparisons by transplant modality are presented. *P* values from aligned rank-transformed ANOVA are presented for differences in protein levels by donor type, sampling time, and their interaction. DD, deceased-donor; LD, living-donor; KT, kidney transplantation; NPX, normalized protein expression.

To further investigate temporal protein release patterns by donor type, we performed ANOVA on aligned rank-transformed data for the 5 proteins upregulated at a *P* value of <0.01 in the pairwise comparison of DD-KT versus LD-KT. Here, levels of HGF, ARTN, OPG, TGF-α, and IL-33 significantly varied by donor type (*P* < 0.001, *P* < 0.001, *P* = 0.005, *P* < 0.001, and *P* < 0.001, respectively) and time postreperfusion (*P* < 0.001 for all). Furthermore, the release of these proteins depended on the combined effect of donor type and elapsed time postreperfusion, as shown by significant interactions (*P* < 0.001 for all; Figure 2).

### Postreperfusion Protein Release and Associations With Short- and Long-term Allograft Dysfunction

The proteins upregulated at a *P* value of <0.01 from the initial screening were consolidated into single individual postreperfusion AUC values on baseline-normalized data, enabling standardized comparisons across proteins with varying release patterns (Figure 2).

Among these proteins, postreperfusion IL-33 and HGF levels showed an association with DGF in the entire study population (ROC AUC 0.77; 95% confidence interval [CI], 0.63-0.91; *P* = 0.0012 and ROC AUC 0.73; 95% CI, 0.59-0.87; *P* = 0.0055, respectively), whereas the remainder of proteins were not associated with this outcome (Table 3). Furthermore, IL-33 levels postreperfusion were significantly higher in DGF patients than in those without DGF (391.55 NPX × minutes [IQR, 286.50–527.30] versus 204.80 NPX × minutes [IQR, 128.20–375.60], *P* = 0.030). There was a trend of increased HGF levels in patients with DGF compared with those without DGF; however, it was not statistically significant (*P* = 0.065; Table 4).

**TABLE 3.** Baseline-adjusted protein levels from 1, 10, and 30 min postreperfusion summarized into AUC and associations with DGF

Protein	ROC AUC	Adjusted *P*	95% CI
IL-33	**0.77**	**0.0012**	**0.63-0.91**
HGF	**0.73**	**0.0055**	**0.59-0.87**
TGF-α	0.66	0.29	0.49-0.83
ARTN	0.59	1.00	0.37-0.81
OPG	0.54	1.00	0.28-0.79

Bold text highlights significant associations.

ARTN, artemin; AUC, area under the curve; CI, confidence interval; DGF, delayed graft function; HGF, hepatocyte growth factor; IL-33, interleukin 33; OPG, osteoprotegerin; ROC, receiver operating characteristic; TGF-α, transforming growth factor alpha.

**TABLE 4.** Mann-Whitney *U* tests by DGF status on the AUC of protein levels from baseline-subtracted data at 1, 10, and 30 min postreperfusion

Protein	DGF (N = 6)	No DGF (N = 57)	*P*
IL-33	391.55 (286.50–527.30)	204.80 (128.20–375.60)	0.030
HGF	110 373.00 (91 281.00–150 703.00)	54 471.00 (7906.00–110 608.00)	0.065
TGF-α	12 087.00 (10 867.00–20 392.00)	10224.00 (4428.00–17 966.00)	0.20
ARTN	23.42 (5.51–32.05)	13.43 (2.83–31.34)	0.47
OPG	3085.50 (943.40–30 639.00)	6593.00 (497.40–12 987.00)	0.78

Protein levels are presented as NPX × minutes in median (first–third quartile).

ARTN, artemin; AUC, area under the curve; DGF, delayed graft function; HGF, hepatocyte growth factor; IL-33, interleukin-33; NPX, normalized protein expression; OPG, osteoprotegerin; TGF-α, transforming growth factor alpha.

To investigate the relationship between postreperfusion protein release and long-term graft dysfunction, multiple linear regression was performed on log-transformed AUCs of baseline-subtracted protein levels and the AUC of eGFR during 1–48 mo posttransplantation in the entire study population. Exclusions from the long-term assessment included 2 DD-KT cases with early graft failure (day 64 and 151) and 1 LD-KT case lost to follow-up (day 69). Remaining graft failures were assigned an eGFR value of 10 mL/min/1.73 m² for the duration of the follow-up, whereas the last recorded eGFR value was used for the deceased patient with a functioning graft (day 939) and the patient lost to late follow-up (day 1100).

Increased postreperfusion HGF release was associated with decreased 4-y allograft function, indicated by the significant negative association between HGF release and the AUC of eGFR (B = –768.20, 95% CI, –1359.03 to –177.37; *P* = 0.012). Conversely, elevated postreperfusion ARTN release was positively associated with the AUC of eGFR (B = 418.96; 95% CI, 48.04-789.87; *P* = 0.028), suggesting an association with increased allograft function during 4 y (Table 5). No relevant associations were observed for TGF-α, OPG, and IL-33 with long-term allograft function.

**TABLE 5.** Log-transformed AUC baseline-adjusted protein levels from 1, 10, and 30 min postreperfusion and associations long-term graft function (AUC of eGFR from 1-, 3-, 6-, 12-, 24-, 36- to 48-mo posttransplantation)

Protein	Unstandardized coefficient B	Standardized coefficient β	*t* value	*P*	95% CI
Constant	4331.74	–	2.43	0.019	735.72 to 7927.77
HGF	**–768.20**	**–0.58**	**–2.62**	**0.012**	**–1359.03 to –177.37**
ARTN	**418.96**	**0.44**	**2.28**	**0.028**	**48.04 to 789.87**
TGF-α	418.39	0.16	0.76	0.45	–694.99 to 1531.77
IL-33	–90.67	–0.04	–0.24	0.81	–842.76 to 661.41
OPG	–64.85	–0.05	–0.31	0.76	–488.20 to 358.49

Bold text highlights significant associations.

ARTN, artemin; AUC, area under the curve; CI, confidence interval; eGFR, estimated glomerular filtration rate; HGF, hepatocyte growth factor; IL-33, interleukin 33; OPG, osteoprotegerin; TGF-α, transforming growth factor alpha.

### Dynamics of Immediate HGF Release in Relation to CIT

**FIGURE 3.**
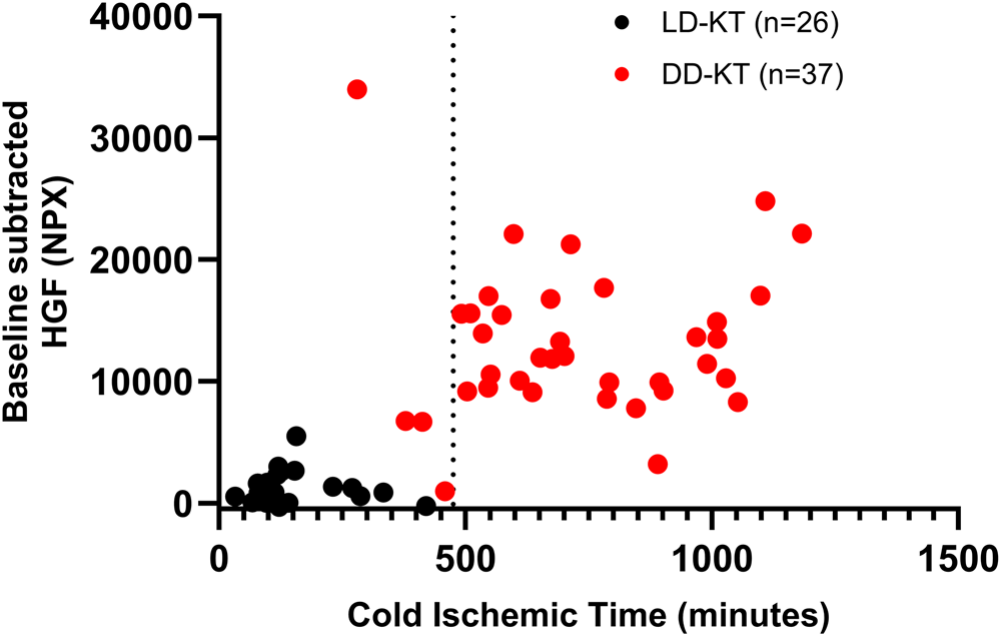
Cold ischemic time and immediate postreperfusion HGF release. Scatter plot of HGF levels by cold ischemic time at 1 min postreperfusion. A vertical dotted line indicates 475.5 min of cold ischemic time. DD, deceased-donor; HGF, hepatocyte growth factor; KT, kidney transplantation; LD, living-donor; NPX, normalized protein expression.

We limited the analysis of protein-CIT associations with HGF to avoid the risk of multiple comparisons and false-positive findings. HGF was selected on the basis of its strong and sustained release pattern in DD-KT, its biological relevance as a marker of ischemic injury, and its consistent association with both DGF and long-term graft dysfunction. A K-means clustering (*k* = 2) identified 2 significantly distinct clusters of HGF levels and elapsed CIT with cluster centers of 1943.0 and 14 580.2 NPX for HGF, along with 244.4 and 743.5 min for CIT (*P* < 0.001 for both). Dichotomization of the study population by cluster membership was followed by an ROC analysis on this dichotomous parameter (membership of the higher CIT cluster) and CIT. Youden’s J Statistic indicated an approximated cutoff of 475.5 min of elapsed CIT (97% sensitivity, 91% specificity) for increased HGF release at 1 min postreperfusion (Figure 3).

## DISCUSSION

In this prospective cohort study of 63 consecutive transplant recipients, we profiled 92 inflammation-associated proteins in venous blood immediately after allograft reperfusion. By correlating these early, inflammatory signatures with allograft function both in the early postoperative period and across a 4-y follow-up, we identified distinct and previously unrecognized protein release patterns that varied by donor type and were linked to divergent clinical outcomes.

In DD-KT, the protein expression profile was characterized by an immediate postreperfusion surge in HGF, 4E-BP1, ARTN, and OPG, followed within 30 min by an early release of IL-33 and TGF-α (Figure 1). Notably, the release of these proteins exhibited no significant difference between preservation methods among DD kidneys (**Table S2, SDC,** https://links.lww.com/TP/D323). Among these proteins, HGF stood out for its pronounced and sustained postreperfusion release, associated with both DGF and long-term dysfunction (Tables 3–5). Known as a pleiotropic cytokine, HGF is recognized for its roles in promoting cell viability, proliferation, and migration, as well as exhibiting anti-inflammatory properties.^12,13^ This raises the question of why HGF release, in the context of IRI during DD-KT, correlates inversely with transplant outcomes.

In models of acute kidney injury, HGF signaling has previously been shown to be upregulated after ischemic or toxic stress,^14,15^ supporting its role in the local response to injury. Interestingly, in conditions such as myocardial infarction and solid tumors, hypoxia-induced injury is known to trigger systemic HGF release.^16,17^ Myocardial infarction, which shares similarities with transplant IRI, has been shown in both clinical and preclinical studies to prompt early systemic HGF release within hours of the ischemic event.^17-19^ Similarly, a previous study on murine renal IRI demonstrated a substantial rise in plasma HGF levels as early as 1 h postreperfusion.^20^ In this study, we are the first to document an immediate and pronounced local release of HGF in DD kidney allografts, enabled by direct sampling from the transplant vein postreperfusion. A plausible explanation for this rapid HGF surge on reperfusion may lie in HGF’s high affinity to heparan sulfate in the extracellular matrix, components of the endothelial and epithelial glycocalyx.^21^

The glycocalyx, a 3-dimensional structure rich in heparan sulfate, glycoproteins, and glycosaminoglycans, confers antithrombotic and anti-inflammatory properties and acts as a reservoir for numerous bioactive mediators, including HGF.^6,22,23^ However, under ischemic stress, the glycocalyx is degraded by upregulation of heparanase and metalloproteinase activity, leading to exposure of endothelial cell surfaces and the release of matrix-bound regulators.^24^ The breakdown of the glycocalyx erodes its protective functions, facilitating a thrombo-inflammatory response, an effect we previously demonstrated in DD kidneys on reperfusion.^8^

In our study, we observed a rapid release of HGF on reperfusion, particularly beyond a critical CIT threshold of 475.5 min (Figure 3). Given the high affinity of HGF for heparan sulfate proteoglycans, we hypothesize that its rapid release on reperfusion in DD kidneys may partly reflect glycocalyx disruption. Interestingly, this threshold aligns with our previously observed 522.4-min cutoff for IRI-induced thromboinflammation, marked by sC5b-9 expression.^8^

Taken together, the parallel time-linked changes in HGF and sC5b-9 point to, but do not establish, a potential association between prolonged ischemia, glycocalyx disruption, and thromboinflammation.

Unlike HGF, ARTN exhibited a transient increase after reperfusion and was not associated with adverse allograft outcomes in this cohort. Although the biological role of ARTN in the context of IRI remains incompletely understood, no clear link to graft dysfunction was observed in this setting.

Alongside HGF, we observed an early increase of IL-33 and TGF-α within 30 min postreperfusion in DD-KT, suggesting a secondary wave of injury-related cytokine release. IL-33, a potent nuclear alarmin stored in endothelial and epithelial cells, is known to be released by necrotic cells under various conditions such as trauma, infection, and renal IRI.^25-27^ Although previous studies have reported elevated IL-33 levels in urine or systemic blood hours after transplantation, correlating with prolonged CIT,^28^ our data show that IL-33 release occurs within the first 10 min postreperfusion locally in the transplant vein. Importantly, early IL-33 elevation was associated with the development of DGF (Tables 3 and 4), reinforcing its role as an early marker of ischemic stress and cellular injury in the context of DD-KT.

TGF-α was similarly elevated early postreperfusion in DD-KT. Although less well characterized in kidney IRI, increased TGF-α expression has been implicated in ischemic acute kidney injury, where it correlates with worsened renal outcomes.^29^ The co-release of IL-33 and TGF-α may therefore reflect a synergistic injury response, possibly amplifying inflammation and cell stress during reperfusion, although this warrants further mechanistic investigation.

Beyond cytokines, we also observed a rapid increase in 4E-BP1 levels within 1 min postreperfusion in DD-KT. Although 4E-BP1 plays a well-documented role in translational control under stress conditions,^30,31^ its early increase during reperfusion in DD-KT is a novel finding. To date, no previous studies have addressed its role in transplant IRI, and the functional implications of this transient response remain unclear.

In contrast to the injury-associated cytokine surge observed in DD-KT, LD-KT displayed a different immune profile, characterized by a shift toward anti-inflammatory and regulatory signaling. Most notably, we observed an immediate release of TRANCE, followed by a delayed expression of IL-6, LIF, FGF-23, MCP-4, and ADA (Figure 1). Interestingly, although TRANCE was immediately released in LD kidneys, DD kidneys exhibited a concurrent release of OPG, a protein known to counteract TRANCE as an inhibitory decoy receptor.^32^ Although data on TRANCE in IRI are limited, preclinical data on ischemic stroke have associated TRANCE signaling with a reduced extent of infarction,^33^ whereas clinical evidence has correlated OPG levels to ischemic stroke severity.^34^ This reciprocal TRANCE-OPG expression pattern may reflect divergent immune-modulatory pathways between transplants of different donor types.

Beyond the immediate TRANCE-OPG dynamics, IL-6 and LIF emerged as key components of the LD-KT cytokine profile. Both cytokines belong to the IL-6 family, exhibiting dual pro- and anti-inflammatory roles. IL-6, traditionally associated with proinflammatory responses in the allogeneic transplant setting,^35^ has also demonstrated protective effects during IRI. Notably, early IL-6 upregulation within 30 min postreperfusion has been previously observed in 1 clinical study involving LD-KT patients.^36^ Complementing these findings, preclinical experiments from the same study demonstrated that IL-6 depletion exacerbates IRI-induced injury. Similar observations have also been made in preclinical models of IRI in the liver and heart,^37,38^ further supporting the role of IL-6 as a critical mediator in mitigating IRI across different organ systems. Furthermore, LIF and FGF-23 appear to reinforce the regulatory shift observed in LD-KT. LIF, for instance, promotes endogenous immune regulation by promoting regulatory T-cell differentiation and suppressing proinflammatory TH-17 T cells,^39^ whereas FGF-23, as demonstrated in preclinical models, mitigates IRI through anti-inflammatory mechanisms when administered exogenously.^40^ These synergistic effects align with the broader anti-inflammatory milieu that may characterize LD-KT.

ADA and MCP-4, typically recognized for their proinflammatory roles,^41,42^ were also upregulated in LD-KT, although their precise functions in this context remain unclear. Although it is possible that ADA and MCP-4 could exhibit context-dependent roles similar to the duality observed in IL-6, LIF, and FGF-23, this hypothesis needs further investigation.

We recognize several limitations in our study. As an exploratory, hypothesis-generating investigation, it is limited by its single-center design and moderate sample size, which may constrain statistical power and generalizability. The study population was restricted to include DD kidneys from only donation-after-brain-death donors, which may reduce transferability to donation-after-circulatory-death. Analyses were also confined to a predefined panel of 92 inflammatory proteins, potentially excluding other relevant markers involved in the IRI response. Although orthogonal validation was not performed, the proteomic platform applied has been analytically validated for specificity, sensitivity, and reproducibility in plasma and serum samples. Our key observations of HGF and IL-33 are consistent with independent studies that confirm the presence of these proteins using orthogonal assays.^43,44^ Taken together, this exploratory study provides a well-characterized clinical cohort to identify candidate markers and generate hypotheses for future validation.

Importantly, by sampling directly from the transplant vein, our approach enabled a transplant-specific characterization of inflammatory responses with greater resolution than systemic biomarker analysis allows. Baseline normalization further accounted for pretransplant inflammatory conditions, ensuring that postreperfusion changes reflected IRI-driven responses. Additionally, potential confounding from immunosuppressants and their timing relative to sampling was likely reduced by adherence to a uniform standard of care protocol. To strengthen analytical rigor, we applied multiple testing correction and tiered significance thresholds, allowing us to prioritize the most robust markers for outcome analyses. These methodological considerations increase the robustness and biological interpretability of the observed donor type-specific inflammatory profiles in DD-KT and LD-KT, as well as their potential relevance for graft outcome prediction.

In conclusion, this study provides important insights into the early inflammatory dynamics after IRI during KT. By applying proteomic profiling in a well-characterized cohort with extended 4-y follow-up, we identified donor type-specific allograft immune signatures with potential relevance for allograft outcomes. These findings highlight the value of early protein profiling in KT to refine risk stratification and guide future biomarker validation efforts.

## Supplementary Material


