# Supplementary material for: Early Postreperfusion Proteomics Reveal Divergent Inflammatory Responses in Kidney Transplantation With Implications on Outcomes
Source: Transplantation. 2025 Oct 31;110(3):e680–8. doi: 10.1097/TP.0000000000005561 (PMC12908640; doi:10.1097/TP.0000000000005561)
Supplement: Supplementary file 1 [file tpa-110-e680-s001.pdf]

Supplements

1 Minute Post-Reperfusion		
Protein	Mean Rank Difference (DD - LD)	Adjusted P Value
**HGF**	**30,85**	**<0,000001**
**ARTN**	**23,9**	**0,000004**
**OPG**	**23,84**	**0,000004**
4E-BP1	16,77	0,021703
TRANCE	-16,08	0,038913
IL17C	-15,55	0,059716
Flt3L	13,36	0,290108
IFNgamma	-13,23	0,310563
TNFB	-13,16	0,319533
CXCL10	-13,1	0,328597
TWEAK	13,03	0,337747
MCP2	-12,25	0,500741
IL12B	-11,98	0,55696
IL10RA	-11,49	0,662297
CCL28	11,4	0,680345
CXCL9	-11,33	0,694046
MCP4	-11,07	0,746164
FGF23	-11,07	0,746164
VEGFA	-11	0,750575
IL20RA	-10,9	0,762635
CCL11	-10,71	0,796493
CST5	-10,67	0,798582
CSF1	10,61	0,809236
IL-33	10,48	0,828246
FGF5	10,12	0,878786
CD5	-9,496	0,94634
SCF	-9,43	0,95068
TNF	-9,365	0,95225
SLAMF1	-9,332	0,952484
IL24	9,332	0,952484
TNFRSF9	-9,234	0,956537
TRAIL	9,168	0,958937
IL20	-8,546	0,986033
SIRT2	7,924	0,996775
STAMBP	7,793	0,997522
IL10RB	-7,728	0,997714
IL10	7,662	0,997888
IL17A	-7,367	0,998973
MMP10	-7,204	0,999346
CCL19	-7,138	0,999397
IL18	-6,876	0,999719
CD8A	-6,811	0,99974
IL2	-6,68	0,999798
GDNF	-6,614	0,999813
CCL4	-6,516	0,999835
LIFR	-6,549	0,999835
CASP8	6,221	0,999933
IL13	-6,058	0,999955
IL-6	-5,959	0,999965
CCL3	-5,567	0,999992
DNER	-5,436	0,999995
MCP1	5,174	0,999998
ST1A1	5,043	0,999999
IL1_α	-4,65	>0,999999
FGF21	4,388	>0,999999
FGF19	-4,453	>0,999999
IL22RA1	-4,126	>0,999999
PDL1	-3,667	>0,999999
uPA	3,536	>0,999999
CXCL5	-3,471	>0,999999
CD40	-3,536	>0,999999
CD244	-3,274	>0,999999
CDCP1	-3,012	>0,999999
IL7	-2,816	>0,999999
LAP_TGFbeta1	3,078	>0,999999
AXIN1	3,143	>0,999999
CXCL1	2,816	>0,999999
CCL25	-2,947	>0,999999
CX3CL1	-3,078	>0,999999
CCL20	3,143	>0,999999
IL8	0,9168	>0,999999
MCP3	-1,834	>0,999999
CXCL11	2,096	>0,999999
IL2RB	-1,539	>0,999999
OSM	2,161	>0,999999
TSLP	-1,539	>0,999999
CD6	0,1965	>0,999999
TGF-α	1,441	>0,999999
TNFSF14	-0,5894	>0,999999
MMP1	1,113	>0,999999
IL15RA	-1,834	>0,999999
IL18R1	-1,113	>0,999999
BetaNGF	-1,735	>0,999999
CCL23	-2,292	>0,999999
CXCL6	-2,292	>0,999999
ENRAGE	1,212	>0,999999
IL4	-2,259	>0,999999
LIF	0,9168	>0,999999
NRTN	0,4912	>0,999999
NT3	0,5894	>0,999999
IL5	1,637	>0,999999
ADA	0,06549	>0,999999

10 Minutes Post-Reperfusion		
Protein	Mean Rank Difference (DD - LD)	Adjusted P Value
**HGF**	**29,73**	**<0,000001**
**TGF-α**	**27,72**	**<0,000001**
**IL-33**	**19,54**	**0,001128**
TNFB	-14,38	0,145715
IL18	-14,24	0,158761
IL20RA	-13,51	0,254906
MCP2	-13,04	0,338524
MCP4	-12,9	0,361685
IL17C	-12,57	0,424709
CCL4	-12,43	0,45417
FGF23	-12,23	0,495668
IFNgamma	-11,9	0,569465
CXCL10	-11,7	0,612216
ARTN	11,6	0,627078
CCL3	-11,16	0,725188
TNF	-11,03	0,749336
ADA	-10,76	0,795856
LIFR	-10,62	0,81657
IL10RB	-10,49	0,839307
VEGFA	-10,36	0,857581
CD244	-9,886	0,910565
CXCL9	-9,954	0,910565
IL10RA	-9,853	0,910565
CCL19	-9,954	0,910565
TNFRSF9	-9,954	0,910565
IL15RA	-9,752	0,917031
CX3CL1	-8,881	0,980358
DNER	-8,747	0,984159
SCF	-8,68	0,98534
GDNF	-8,345	0,992368
CDCP1	-8,345	0,992368
LAP_TGFbeta1	-8,278	0,992396
CST5	-8,278	0,992396
ENRAGE	-8,278	0,992396
NT3	-8,278	0,992396
IL-6	-8,144	0,992608
CCL23	-8,077	0,992608
CXCL6	-8,144	0,992608
CCL11	-7,943	0,994043
CXCL5	-7,675	0,996691
CD5	-7,675	0,996691
TWEAK	7,406	0,998025
PDL1	-7,339	0,998157
IL12B	-7,272	0,998276
IL7	-7,205	0,998303
IL18R1	-6,87	0,999336
IL10	6,736	0,9995
TRANCE	-6,602	0,999603
SLAMF1	-5,965	0,999957
TNFSF14	-5,932	0,999957
LIF	-5,965	0,999957
CD8A	-5,798	0,99996
SIRT2	-5,731	0,999961
CCL25	-5,664	0,999962
IL2RB	-5,563	0,999966
TSLP	-5,496	0,999966
CASP8	-5,429	0,999966
OPG	5,329	0,999972
IL20	-5,128	0,999983
BetaNGF	-4,96	0,999989
IL17A	-4,524	0,999997
CXCL11	-4,591	0,999997
AXIN1	-4,524	0,999997
CXCL1	-4,189	0,999997
CD6	-4,29	0,999997
FGF21	4,524	0,999997
IL22RA1	-4,29	0,999997
IL13	-4,357	0,999997
CD40	-3,988	0,999997
FGF19	-4,457	0,999997
STAMBP	-3,653	0,999997
MMP10	-3,385	0,999999
CCL20	-2,715	>0,999999
IL4	2,145	>0,999999
NRTN	2,178	>0,999999
IL8	-1,91	>0,999999
MCP3	-1,274	>0,999999
uPA	-1,642	>0,999999
MCP1	-0,9049	>0,999999
TRAIL	-1,441	>0,999999
IL1_α	1,575	>0,999999
OSM	1,274	>0,999999
IL2	0,9719	>0,999999
FGF5	0,7038	>0,999999
MMP1	-0,6368	>0,999999
IL24	-1,106	>0,999999
Flt3L	1,441	>0,999999
CCL28	0,3351	>0,999999
ST1A1	1,642	>0,999999
IL5	-1,173	>0,999999
CSF1	-1,307	>0,999999
4E-BP1	0,03351	>0,999999

30 Minutes Post-Reperfusion		
Protein	Mean Rank Difference (DD - LD)	Adjusted P Value
**IL-33**	**20,41**	**0,000417**
**HGF**	**19,47**	**0,001256**
**TGF-α**	**18,47**	**0,003758**
IL-6	-16,92	0,017329
LIF	-16,59	0,02306
FGF23	-16,32	0,029438
ADA	-16,19	0,032781
MCP4	-15,72	0,048596
IFNgamma	-15,45	0,060077
IL18	-14,18	0,157799
IL7	-13,84	0,196716
IL12B	-13,77	0,20351
IL17C	-13,61	0,222256
CXCL5	-13,44	0,247476
CXCL10	-13,3	0,266135
CCL23	-13,17	0,285638
MCP2	-13,17	0,285638
TNFB	-13,04	0,299598
CXCL9	-12,84	0,336066
IL20RA	-12,6	0,371449
SIRT2	-12,63	0,371449
LAP_TGFbeta1	-12,57	0,376761
NRTN	-12,33	0,417231
SCF	-12,23	0,437857
CXCL6	-12,1	0,462239
CXCL1	-11,76	0,528513
NT3	-11,76	0,528513
CD5	-11,63	0,548741
DNER	-11,63	0,548741
CCL19	-11,43	0,586767
VEGFA	-11,09	0,656864
CD8A	-11,03	0,665839
CST5	-10,76	0,718131
CCL4	-10,62	0,740191
IL15RA	-10,32	0,790544
GDNF	-10,29	0,791636
CDCP1	-10,26	0,791636
CCL3	-10,29	0,791636
IL22RA1	-10,19	0,792168
IL10RB	-10,02	0,819453
CXCL11	-9,954	0,825114
BetaNGF	-9,786	0,843831
CX3CL1	-9,752	0,846485
TNFRSF9	-9,618	0,861385
CASP8	-9,484	0,87523
IL20	-9,116	0,914173
ENRAGE	-9,015	0,922378
TNFSF14	-8,948	0,924879
TNF	-8,814	0,933574
ARTN	8,613	0,945489
CD244	-8,345	0,961153
CD6	-8,278	0,961153
IL10RA	-8,345	0,961153
MMP1	-8,278	0,961153
LIFR	-8,278	0,961153
IL18R1	-8,345	0,961153
TRANCE	-8,077	0,961153
CCL20	-8,211	0,961153
CSF1	-7,742	0,971269
SLAMF1	-7,675	0,971447
AXIN1	-7,205	0,986218
STAMBP	-7,138	0,986218
CD40	-7,004	0,987711
uPA	-6,267	0,997195
MCP1	-6,267	0,997195
IL17A	-6,032	0,997527
FGF21	5,932	0,997698
PDL1	-5,932	0,997698
MCP3	-5,664	0,998024
OSM	-4,725	0,998024
IL2	-5,061	0,998024
CCL11	-5,597	0,998024
FGF5	-5,63	0,998024
MMP10	-5,195	0,998024
IL10	5,597	0,998024
4E-BP1	-5,396	0,998024
IL4	-5,228	0,998024
CCL25	-4,524	0,998024
TWEAK	-4,591	0,998024
ST1A1	-4,524	0,998024
TRAIL	-3,921	0,998149
IL13	-2,916	0,999059
FGF19	-3,418	0,999059
IL5	-3,351	0,999059
Flt3L	-2,514	0,999318
TSLP	-2,078	0,999489
CCL28	-2,044	0,999489
IL8	0,7373	0,999862
OPG	-1,039	0,999862
IL2RB	-0,8043	0,999862
IL1_α	0,4357	0,999862
IL24	-0,5362	0,999862

Table S1. Assessed baseline subtracted protein levels by mean rank difference of compared kidney transplant cases by donor type and corresponding *P* values for sampling time points at 1, 10, and 30 minutes post-reperfusion. Bold red text indicates a *P* value less than 0.01. Blue text denotes *P* values less than 0.05. DD=Deceased-donor, LD=Living-donor.

1 Minute Post-Reperfusion		
Protein	Mean Rank Difference (SCS - HMP)	Adjusted P Value
IL8	-9,194	0,605992
TWEAK	11,55	0,089001
FGF23	-10,99	0,156839
MMP10	-10,99	0,156839
TGFα	9,194	0,605992
IL18	-8,858	0,702002
TRANCE	-8,633	0,762749
TNFRSF9	-8,409	0,817593
Flt3L	7,736	0,939629
IL2	-7,68	0,940928
CXCL1	-7,624	0,948901
TNFB	-7,512	0,958621
IL17A	-7,288	0,974945
IL20	6,952	0,988866
OSM	-6,839	0,992422
FGF5	6,727	0,994438
OPG	6,615	0,995974
CXCL9	-6,503	0,997127
IL12B	-6,391	0,997979
CD5	-6,111	0,999229
CD244	-6,055	0,999419
IL7	-6,055	0,999419
PDL1	-5,83	0,999753
TNFSF14	-5,662	0,999863
CXCL5	-5,27	0,999986
NT3	-5,27	0,999986
SLAMF1	-4,933	0,999998
IL10	-4,821	0,999998
TNF	-4,821	0,999998
IL33	-4,933	0,999998
CCL20	-4,821	0,999998
IL17C	-4,709	0,999999
SCF	-4,709	0,999999
IFNgamma	-4,709	0,999999
VEGFA	-4,148	>0,999999
CD8A	-4,485	>0,999999
MCP3	-1,794	>0,999999
GDNF	0,1682	>0,999999
CDCP1	-2,803	>0,999999
LAP_TGFbeta1	-3,027	>0,999999
uPA	0,3364	>0,999999
IL6	0,4485	>0,999999
MCP1	-2,691	>0,999999
CXCL11	-1,233	>0,999999
AXIN1	-2,242	>0,999999
TRAIL	0	>0,999999
IL20RA	-1,065	>0,999999
CST5	-2,355	>0,999999
IL2RB	-1,514	>0,999999
IL1_α	-3,027	>0,999999
TSLP	-2,355	>0,999999
CCL4	-2,355	>0,999999
CD6	0,2242	>0,999999
MCP4	-3,139	>0,999999
CCL11	-1,345	>0,999999
IL10RA	-2,13	>0,999999
MMP1	-3,364	>0,999999
LIFR	-1,906	>0,999999
FGF21	2,018	>0,999999
CCL19	-1,345	>0,999999
IL15RA	-1,009	>0,999999
IL10RB	-4,261	>0,999999
IL22RA1	-4,205	>0,999999
IL18R1	-4,597	>0,999999
BetaNGF	-4,261	>0,999999
HGF	2,242	>0,999999
IL24	2,635	>0,999999
IL13	0,1682	>0,999999
ARTN	2,915	>0,999999
CCL23	1,009	>0,999999
CCL3	-4,092	>0,999999
CXCL6	-3,588	>0,999999
CXCL10	-0,2242	>0,999999
4E-BP1	1,121	>0,999999
SIRT2	2,579	>0,999999
CCL28	3,98	>0,999999
DNER	-3,924	>0,999999
ENRAGE	0,5045	>0,999999
CD40	-3,924	>0,999999
FGF19	-0,6727	>0,999999
IL4	-0,7288	>0,999999
LIF	3,476	>0,999999
NRTN	2,018	>0,999999
MCP2	-2,467	>0,999999
CASP8	1,906	>0,999999
CCL25	0	>0,999999
CX3CL1	1,458	>0,999999
ST1A1	1,009	>0,999999
STAMBP	-0,2242	>0,999999
IL5	2,523	>0,999999
ADA	-4,261	>0,999999
CSF1	2,355	>0,999999

10 Minutes Post-Reperfusion		
Protein	Mean Rank Difference (SCS - HMP)	Adjusted P Value
IL8	-10,09	0,346873
TRANCE	-9,53	0,507512
CCL20	-9,194	0,610093
ADA	-8,633	0,774362
CXCL1	-8,521	0,800361
IL10RA	-8,409	0,817485
FGF23	-8,409	0,821208
IL2	-8,129	0,873734
TNFB	-7,848	0,92454
IL17A	-7,736	0,937578
TNF	-7,624	0,948901
IL1_α	-7,512	0,955533
CDCP1	-7,288	0,974945
IL18	-6,952	0,989815
IL10	-6,615	0,996518
TNFRSF9	-6,615	0,996518
LAP_TGFbeta1	-6,391	0,998291
CXCL9	-6,279	0,99883
CD8A	-6,167	0,999211
NT3	-6,111	0,999231
IL7	-6,055	0,999419
CXCL5	-5,942	0,999618
TRAIL	-5,718	0,999861
SLAMF1	-5,606	0,999914
IFNgamma	-5,606	0,999914
CD244	-5,494	0,999939
TNFSF14	-5,27	0,999978
IL12B	-5,158	0,999987
IL5	-5,158	0,999987
AXIN1	-5,045	0,999992
MMP10	-5,045	0,999992
CSF1	-5,045	0,999992
CCL3	-4,821	0,999997
IL4	-4,709	0,999998
CCL25	-4,709	0,999998
CCL4	-4,485	0,999999
IL18R1	-4,597	0,999999
DNER	-4,597	0,999999
IL33	-4,597	0,999999
VEGFA	-2,579	>0,999999
MCP3	-2,971	>0,999999
GDNF	-3,756	>0,999999
OPG	0,2242	>0,999999
uPA	-3,812	>0,999999
IL6	-2,915	>0,999999
IL17C	-4,092	>0,999999
MCP1	-4,373	>0,999999
CXCL11	-0,7848	>0,999999
IL20RA	-2,411	>0,999999
CST5	-2,691	>0,999999
IL2RB	-2,242	>0,999999
OSM	-3,252	>0,999999
TSLP	-0,6167	>0,999999
CD6	-2,355	>0,999999
SCF	-2,018	>0,999999
TGFα	2,018	>0,999999
MCP4	-3,252	>0,999999
CCL11	-2,915	>0,999999
FGF5	-0,2242	>0,999999
MMP1	-3,364	>0,999999
LIFR	-0,897	>0,999999
FGF21	0,897	>0,999999
CCL19	-4,261	>0,999999
IL15RA	-4,036	>0,999999
IL10RB	-3,364	>0,999999
IL22RA1	-3,364	>0,999999
PDL1	-3,252	>0,999999
BetaNGF	-4,373	>0,999999
HGF	3,588	>0,999999
IL24	-2,355	>0,999999
IL13	-2,074	>0,999999
ARTN	0,953	>0,999999
CCL23	-0,7848	>0,999999
CD5	-2,355	>0,999999
Flt3L	-0,2242	>0,999999
CXCL6	-3,476	>0,999999
CXCL10	-2,691	>0,999999
4E-BP1	-0,1121	>0,999999
IL20	1,962	>0,999999
SIRT2	-4,148	>0,999999
CCL28	-4,373	>0,999999
ENRAGE	-2,579	>0,999999
CD40	-2,13	>0,999999
FGF19	-1,682	>0,999999
LIF	-2,635	>0,999999
NRTN	-4,148	>0,999999
MCP2	-3,476	>0,999999
CASP8	-1,682	>0,999999
CX3CL1	-0,897	>0,999999
TWEAK	1,345	>0,999999
ST1A1	-2,579	>0,999999
STAMBP	-3,924	>0,999999

30 Minutes Post-Reperfusion		
Protein	Mean Rank Difference (SCS - HMP)	Adjusted P Value
**CXCL1**	**-12,89**	**0,017583**
**ADA**	**-12,22**	**0,040775**
**IL7**	**-12,11**	**0,046124**
IL8	-10,88	0,171214
AXIN1	-10,54	0,230752
CCL20	-10,2	0,303449
CD244	-10,09	0,328474
TNFSF14	-10,09	0,328474
FGF23	-9,755	0,413322
IL10RA	-9,642	0,433563
TRANCE	-9,642	0,438301
LAP_TGFbeta1	-9,306	0,536511
CXCL5	-9,306	0,536511
SLAMF1	-9,194	0,562524
STAMBP	-9,082	0,5929
TNFB	-9,082	0,5929
SIRT2	-8,97	0,618219
CCL25	-8,858	0,647844
CXCL6	-8,633	0,710006
IL18	-8,409	0,768065
MMP1	-8,073	0,846052
TNF	-8,073	0,846052
IL17A	-7,961	0,861972
CCL4	-7,961	0,861972
MCP2	-7,961	0,861972
ST1A1	-7,905	0,861972
CASP8	-7,848	0,868718
TNFRSF9	-7,848	0,868718
TRAIL	-7,736	0,882211
IL22RA1	-7,736	0,882211
IL17C	-7,512	0,908643
CXCL9	-7,512	0,909151
BetaNGF	-7,344	0,925406
MCP4	-7,176	0,947097
CCL3	-7,176	0,947097
CCL28	-7,176	0,947097
IL2	-7,008	0,950864
4E-BP1	-7,064	0,950864
CXCL11	-6,952	0,956512
CD8A	-6,615	0,977147
CDCP1	-6,615	0,977147
TSLP	-6,615	0,977147
NT3	-6,391	0,983692
IL20RA	-6,223	0,98785
ENRAGE	-6,279	0,98785
IFNgamma	-6,279	0,98785
CST5	-6,055	0,991441
NRTN	-5,998	0,991441
VEGFA	-5,942	0,99174
CD5	-5,942	0,99174
SCF	-5,606	0,996639
CCL19	-5,606	0,996639
PDL1	-5,606	0,996639
IL10	-5,606	0,996639
IL5	-5,494	0,996639
IL15RA	-5,382	0,996719
CX3CL1	-5,27	0,997356
MCP3	-4,933	0,998574
CD6	-4,933	0,998574
CCL11	-4,933	0,998574
CCL23	-5,045	0,998574
DNER	-5,045	0,998574
CSF1	-5,045	0,998574
MCP1	-4,597	0,999043
IL18R1	-4,597	0,999043
CD40	-4,597	0,999043
GDNF	-4,148	0,999116
IL10RB	-4,373	0,999116
MMP10	-4,373	0,999116
CXCL10	-4,261	0,999116
HGF	4,036	0,999177
TGFα	3,812	0,999507
LIFR	-3,7	0,999539
FGF5	-3,364	0,99972
IL12B	-3,476	0,99972
IL4	-3,252	0,99972
LIF	-3,364	0,99972
OPG	-1,009	0,999931
uPA	-2,691	0,999931
IL6	-2,467	0,999931
IL2RB	-2,355	0,999931
IL1_α	-2,579	0,999931
OSM	-2,13	0,999931
FGF21	2,691	0,999931
IL24	0,1121	0,999931
IL13	-0,4485	0,999931
ARTN	-0,7848	0,999931
Flt3L	-1,57	0,999931
IL20	-1,794	0,999931
IL33	-0,897	0,999931
FGF19	-2,579	0,999931
TWEAK	-2,13	0,999931

Table S2. Assessed baseline subtracted protein levels by mean rank difference of compared deceased-donor kidneys by preservation method and corresponding *P* values for sampling time points at 1, 10, and 30 minutes post-reperfusion. Bold text indicates a *P* value less than 0.05. SCS=Static cold storage, HMP=Hypothermic machine perfusion.
